# A Case of a Newborn With Nemaline Myopathy From Al-Qunfudhah City, Saudi Arabia

**DOI:** 10.7759/cureus.52523

**Published:** 2024-01-18

**Authors:** Bushra M Alghanmi, Manal M Alghanmi, Mohammed R Alhayli, Randa M Taffour, Safeyah M Alghubayshi

**Affiliations:** 1 General Practice, South Al-Qunfudah General Hospital, Al-Qunfudah, SAU; 2 Pediatrics and Neonatal Intensive Care Unit, South Al-Qunfudah General Hospital, Al-Qunfudah, SAU; 3 Pediatric Intensive Care Unit, South Al-Qunfudah General Hospital, Al-Qunfudah, SAU

**Keywords:** hypotonia, nemaline, diagnosis, neonates, myopathy

## Abstract

Nemaline myopathy is a primary skeletal muscle disorder and one of the congenital myopathies. It can be caused by mutations in at least 12 genes, with the *nebulin *(*NEB*) gene being the most common. Here, we present the first case of a neonate with nemaline myopathy from Al-Qunfudhah, Saudi Arabia. A full-term baby boy was delivered via cesarean section due to decreased fetal movement. The baby was covered with a thick meconium stain. He was born with severe distress and underwent an endotracheal tube placement. The baby presented generalized muscle weakness, hypotonia, and areflexia. Examination revealed arthrogryposis, bilateral small chin, undescended testicle, joint deformity, hip dislocation, and clubfoot. Chest examination revealed conducting sound and bilateral equal air entry. Moreover, he experienced bilateral chest wheeze and conducting sound. All laboratory tests were normal, and whole-exome sequencing revealed pathogenic homozygous splice acceptor variant *NEB* gene c.8889+1G˃A. The patient was first suspected to have spinal muscular atrophy as there was no previous nemaline myopathy case reported from Al-Qunfudhah. However, the typical symptoms and genetic sequencing confirmed his condition. As the society in Al-Qunfudhah is known for consanguinity, as in our case, clinicians should identify other types of myopathy as it is expected to occur in further cases.

## Introduction

Nemaline myopathy is a congenital myopathy ranging in severity from severe to milder muscle disorders [1]. Nemaline myopathy describes a group of muscle diseases presenting at birth or early infancy with muscle weakness and hypotonia [2]. Nemaline myopathy may result from mutations in at least 12 genes such as *ACTA1*, *TPM2*, *TPM2*, and *NEB*, and some cases remain molecularly unresolved [2]. The clinical phenotypes of nemaline myopathies are of a wide spectrum, even in individuals with mutations in the same gene or even in the same family [3]. The major symptoms of congenital myopathy are hypotonia and skeletal muscle weakness [4]. The symptoms of nemaline myopathy may present at any point during life, from fetal development to adulthood [5]. Nemaline myopathy is a rare disease with an incidence of 1 in 50,000 live births [6]. Here, we present the first case of a neonate with nemaline myopathy from Al-Qunfudhah.

## Case presentation

A full-term baby boy was born to a 26-year-old mother, gravida 2 para 1, via lower-segment cesarean section due to decreased fetal movement and a history of polyhydramnios in October 2019 at South Al-Qunfudhah General Hospital. The APGAR score at delivery was 1 at one minute. Following intubation, the APGAR score increased to 8 at five minutes. The weight of the baby at birth was 2.2 kg. The baby was covered with a thick meconium stain, and he underwent oronasal suction. Due to severe distress (cyanosis and bradycardia) at birth, he received oxygen without improvement. An ambu bag was administered, without improvement, following which he underwent intubation with improvement. As he failed extubation three times, a tracheostomy was performed.

The baby was suffering from generalized weakness and muscle weakness, hypotonia, and areflexia. He had an unusual look due to arthrogryposis, a small bilateral chin, and an undescended testicle. Chest examination revealed conducting sound and bilateral equal air entry. He had eye discharge and was found to have hip dislocation and clubfoot; hence, he was suspected to have spinal muscular atrophy.

All laboratory tests were normal, including complete blood count, chemistry, creatine kinase, thyroid function test, and septic screening, whereas metabolic screening was 21. On the 16th day of birth, the baby displayed an increase in urea level and positive C-reactive protein but with normal blood culture/sensitivity. A chest examination revealed rhonchi. An ultrasound was done but nerve conduction studies were not done.

Whole-exome sequencing revealed pathogenic homozygous splice acceptor variant *NEB *gene c.8889+1G˃A. The baby was diagnosed with an autosomal recessive nemaline myopathy.

## Discussion

Nemaline myopathy is a primary skeletal muscle disorder with variable clinical presentation and genetic causes. Nemaline myopathy may present similarly to other congenital myopathies [5]. Our patient presented with generalized weakness, muscle weakness, and hypotonia. Although the differential diagnosis for cases with severe hypotonia is broad, at birth, it includes spinal muscular atrophy, type 1 myotonic dystrophy, and other conditions [7,8]. Therefore, the patient was first suspected of having spinal muscular atrophy as it is characterized by proximal muscle weakness, and patients affected with type 1 spinal muscular atrophy experience clinical signs before six months of age and have profound hypotonia. Additionally, spontaneous activity is poor [9].

Therefore, the diagnosis of nemaline myopathy is based on a multidisciplinary approach with careful pathologic, genetic, and clinical correlations [2]. Our patient underwent an ultrasound; muscle MRI is an important contributor to diagnosis and highlights specific characteristics of muscle involvement associated with particular genes [10]. However, the patient showed no improvement despite his laboratory parameters being normal. *Klebsiella *was treated with amikacin.

**Figure 1:**
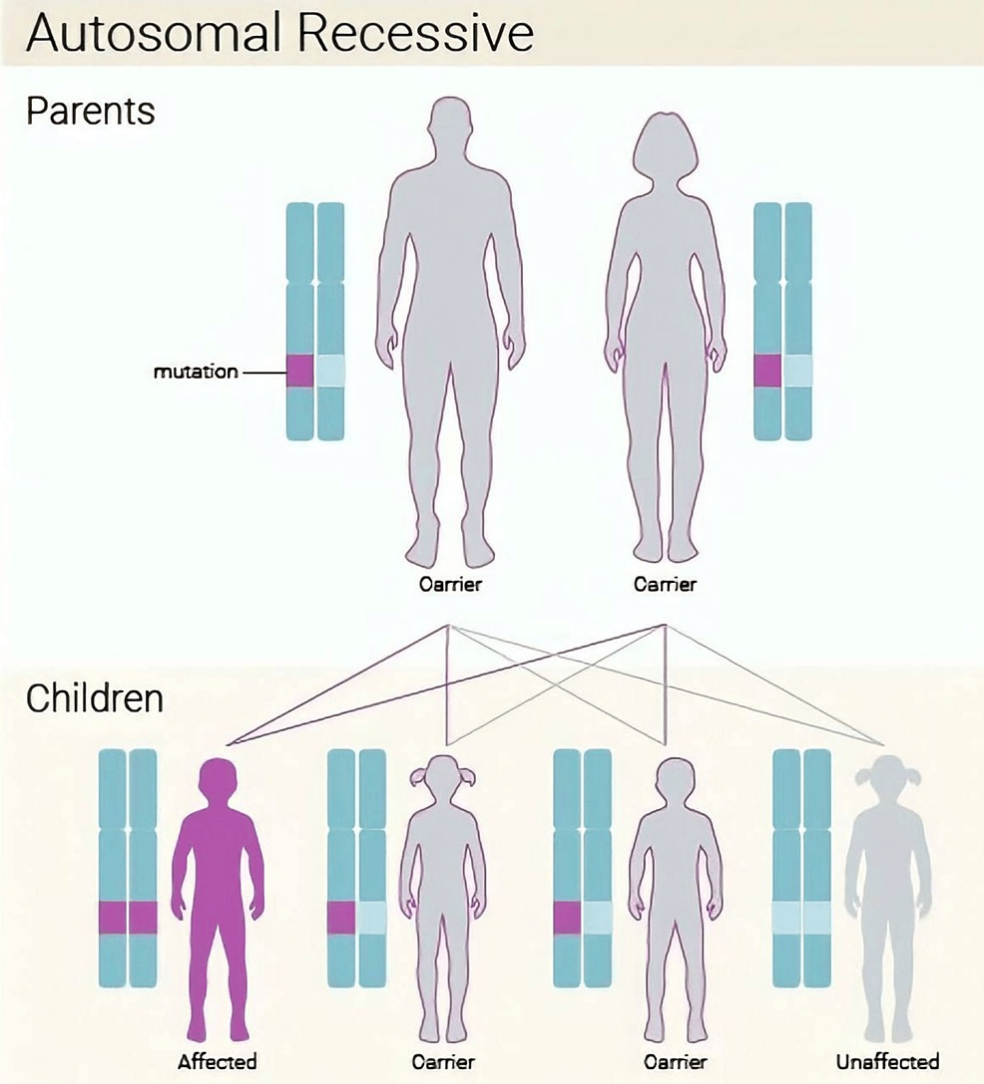
Autosomal recessive pattern in nemaline myopathy. [12].

The accuracy of diagnosis of various rare congenital disorders has been improved by the development of sequencing [11]. The whole-exome sequencing for our patient revealed pathogenic homozygous splice acceptor variant *NEB *gene c.8889+1G˃A; therefore, the baby was diagnosed with an autosomal recessive nemaline myopathy(Figure 1).

Nemaline myopathy may occur as a result of mutations in at least 12 genes [2]. The most commonly causative mutations of nemaline myopathy occur in the gene encoding skeletal muscle nebulin [13]. Therefore, our patient experienced nemaline myopathy due to common mutations of *NEB*. Additionally, the condition was caused by an autosomal recessive gene. *NEB* mutations are commonly recessively inherited [2].

The *NEB* gene had multiple splice sites and triplicate repeat regions, where the most common large variants of the gene are detected [14]. In our patient, there was a homozygous splice acceptor variant of the *NEB *gene. The onset of severe weakness in utero related to nemaline myopathy may lead to fetal akinesia sequence with arthrogryposis; however, presentation at birth involves generalized weakness [1]. This was also noted in our patient. The clinical phenotypes of nemaline myopathy range from severe disease of neonates and onset in utero to mild childhood-onset forms [3].

The onset in our patient seemed to occur in utero as the mother experienced reduced fetal movement in the third trimester. Even though all characteristics of nemaline myopathy were typically present in our patient, he was misdiagnosed at first due to similarities between nemaline myopathy and spinal muscular atrophy, including muscular weakness. This is the first case of nemaline myopathy from Al-Qunfudhah City.

## Conclusions

Nemalin myopathy is one of the rare congenital myopathies, and its main symptoms include hypotonia and generalized weakness. It occurs due to genetic mutations in at least 12 genes, but mutations of *NEB *are the most common causative factor. Our patient was diagnosed with nemaline myopathy by genetic sequencing. Clinicians should identify other forms of myopathy as it is expected that further babies may be present with myopathy, especially as the community is well-known for consanguinity.

The prognosis for the baby was bad, and he was transferred to a private hospital by his parents to complete follow-up, physiotherapy, and supportive management. Parental counseling was done, but screening has not worked yet. They gave birth to a healthy second baby two years ago.
